# The Origin and Nature of Cancer

**Published:** 1873-06

**Authors:** 


### THE ORIGIN AND NATURE OF CANCER.

It has been a source of satisfaction to us to learn that Dr. Woodward, of Washington, D. C., in charge of the United States Army Medical Museum of that city, is engaged in a series of investigations on the origin and nature of cancer, and that the medical profession of this country is thus furnishing one coöperator in the important investigation of the great cancer question, which has beeen studied and discussed again with renewed zeal by the prominent histologists of Europe, and particularly Germany, for the last ten or fifteen years.

Outside of a few verbal statements relating to the contents of a paper read by Dr. Woodward, on this subject, at the last meeting of the American Medical Association, we know nothing of the results of his labors, but are inclined to think they will lead him to the same or similar histological and clinical opinions as are now advocated in Europe by the most prominent investigators, amongst whom Virchow, Haldeier, Billroth, Strisker, Ròster, and others occupy a prominent position.

While these histologists, in their efforts to establish a unique anatomical basis and mode of development, characterizing and defining cancer from other morbid growths, have not yet succeeded—while Virchow, Ròster, W. Miller, Forster, etc., contend for the development of the cancer cell, from the connective tissue—Waldeyer, Billroth, Robin, Cornil, and others, favor its development from the different surfaces and glandular epithelia. Their researches leave no doubt that cancer in its various forms is, primarily and strictly, local degeneration; that loose cancer cells, by finding their way sooner or later into tracts of the lymphatics, can originate the same morbid process in other organs and parts of the system, and that the so-called cancerous cachexia is probably more the unavoidable result of the resorption of large quantities of detritus and decaying material, and the effect of pain and depression, than of a specific constitutional change in the condition of the blood.

In consequence of these opinions the prevailing principles now governing, or about to govern the treatment of cancerous growths are:

1. Early removal of the entire mass of the affected organ (even if the larger portion of it should be apparently sound)

within the limits of the healthy tissue around it, with every lymphatic gland showing any marks of infection.

2. Operation by the acute ligature, (ligatura caudens,) or the galvani-cautery, not the knife, in order to avoid the possibility of inoculating the stroma, or cancer cells, into the healthy tissues, which could be done by the knife should it come in contact with such.

Billroth, at the last Congress of German Surgeons, at Berlin, in April last, in a discussion about the propriety of the surgical removal of carcinoma of the tongue, used the follawing emphatic language: "I am fully convinced that the large majority of canceromatous degenerations would be curable if a complete removal of every morbid portion could be accomplished."

We look with impatience for the publication of Dr. Woodward's labors.

